# Supplementary material for: From sequence to enzyme mechanism using multi-label machine learning
Source: BMC Bioinformatics. 2014 May 19;15:150. doi: 10.1186/1471-2105-15-150 (PMC4229970; doi:10.1186/1471-2105-15-150)
Supplement: Additional file 2 — Java code of ml2db. Additional file ml2db_code.tar.gz contains the Java source code to run the multi-label machine learning experiments and save the results to database. The code’s Javadoc is included. [file 1471-2105-15-150-S2.zip › additional file 2/ml2db/ml2db/doc/test/al/utility/neighbourhood/NearestNeighboursByFreqUtilityTest.html]

NearestNeighboursByFreqUtilityTest


---


|  |  |  |
| --- | --- | --- |
| **Overview** \| **Package** \| **Class** \| **Use** \| **Tree** \| **Deprecated** \| **Index** \| **Help** | |  |
| PREV CLASS   **NEXT CLASS** | **FRAMES**    **NO FRAMES**     **All Classes** |  |
| SUMMARY: NESTED \| FIELD \| CONSTR \| METHOD | DETAIL: FIELD \| CONSTR \| METHOD |  |


---


## test.al.utility.neighbourhood Class NearestNeighboursByFreqUtilityTest

```
java.lang.Object
  test.al.utility.neighbourhood.NearestNeighboursByFreqUtilityTest
```

---

``` public class NearestNeighboursByFreqUtilityTest extends java.lang.Object ```

Class

**Version:**
:   13 May 2012

**Author:**
:   Luna De Ferrari luna.deferrari@ed.ac.uk or
    luna.deferrari@st-andrews.ac.uk

---

**Constructor Summary**

`NearestNeighboursByFreqUtilityTest()`


| **Method Summary** | |
| --- | --- |
| `static NearestNeighboursByFreqUtility` | `getNNUtilityTestSet()` |
| `static NearestNeighboursByFreqUtility` | `getNNUtilityTrainSet()` |
| `void` | `testGetFreqToInstancesMap()` |
| `void` | `testGetFreqToInstancesMap2()` |
| `void` | `testGetInstanceToFeatureMap()` |
| `void` | `testGetInstanceToFeatureMap2()` |
| `void` | `testGetLinearNNSearch()`             Test method for `NearestNeighboursByFreqUtility.getLinearNNSearch()` . |

**Methods inherited from class java.lang.Object**

`equals, getClass, hashCode, notify, notifyAll, toString, wait, wait, wait`

**Constructor Detail**

### NearestNeighboursByFreqUtilityTest

```
public NearestNeighboursByFreqUtilityTest()
```


**Method Detail**

### testGetFreqToInstancesMap

```
public void testGetFreqToInstancesMap()
```

---


### testGetFreqToInstancesMap2

```
public void testGetFreqToInstancesMap2()
```

---


### testGetInstanceToFeatureMap

```
public void testGetInstanceToFeatureMap()
```

---


### testGetInstanceToFeatureMap2

```
public void testGetInstanceToFeatureMap2()
```

---


### testGetLinearNNSearch

```
public void testGetLinearNNSearch()
```

:   Test method for
    `NearestNeighboursByFreqUtility.getLinearNNSearch()`
    .

---


### getNNUtilityTestSet

```
public static NearestNeighboursByFreqUtility getNNUtilityTestSet()
```

---


### getNNUtilityTrainSet

```
public static NearestNeighboursByFreqUtility getNNUtilityTrainSet()
```


---


|  |  |  |
| --- | --- | --- |
| **Overview** \| **Package** \| **Class** \| **Use** \| **Tree** \| **Deprecated** \| **Index** \| **Help** | |  |
| PREV CLASS   **NEXT CLASS** | **FRAMES**    **NO FRAMES**     **All Classes** |  |
| SUMMARY: NESTED \| FIELD \| CONSTR \| METHOD | DETAIL: FIELD \| CONSTR \| METHOD |  |


---
